# Supplementary material for: Glutamate spillover in C. elegans triggers repetitive behavior through presynaptic activation of MGL-2/mGluR5
Source: Nat Commun. 2019 Apr 23;10:1882. doi: 10.1038/s41467-019-09581-4 (PMC6478929; doi:10.1038/s41467-019-09581-4)
Supplement: Supplementary file 1 — Supplementary Information [file 41467_2019_9581_MOESM1_ESM.docx]

**Glutamate spillover in *C. elegans* triggers repetitive behavior through presynaptic activation of MGL-2/mGluR5**

Katz et al. **Supplementary Information**

**Supplementary Figures**

**
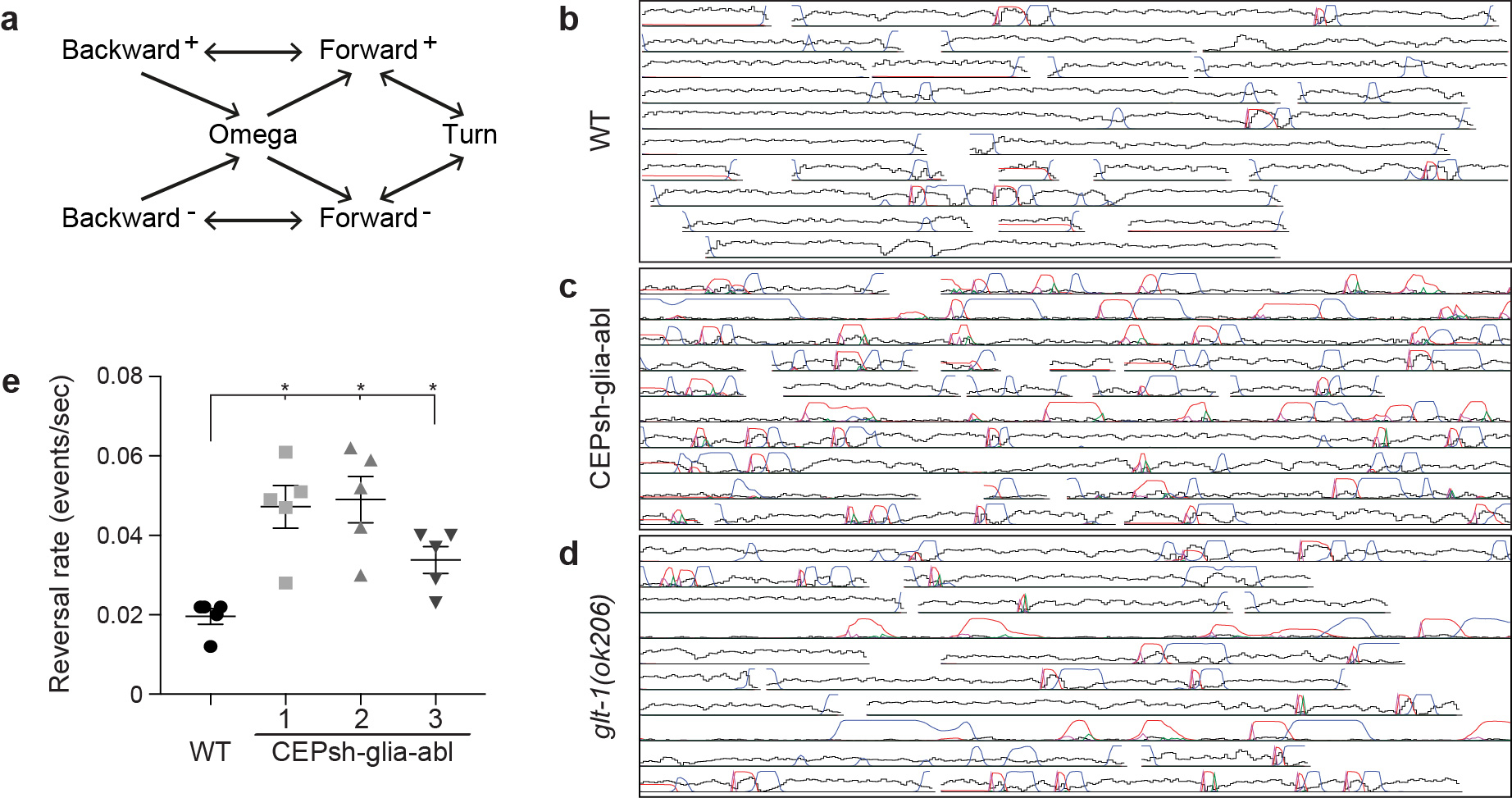
**

**Supplementary Fig. 1. Analysis of reversal behavior.**

**a,** Hidden Markov Model for worm reversals. The model comprises six behavioral states: forward and backward crawling, which are subdivided into two states (labeled + and -; depending on whether motion along the arbitrarily oriented body axis corresponds to forward or backward movement), forward turn, and omega turn. Not all possible transitions are permitted: for instance, the sign (+ or -) must remain the same when the worm reverses, and an omega turn occurs only in the sequence backward crawling → omega turn → forward crawling. **b-d,** Representative tracks analyzed with multiple worm-tracker code. Overlapping tracks in different rows originate from different animals. Tracks in each row are not necessarily from the same animal. Black lines, instantaneous speed. Colored lines: estimated probability of different states and state transitions; red, backward crawling state; blue, turning state (forward turn or omega turn); magenta, forward crawling to backward crawling (reversal); green, backward crawling to forward crawling. (**b**) Wild type (WT), (**c**) CEPsh glia-ablated, (**d**) *glt-1(ok206)* animals. **e,** CEPsh glia ablation results in enhanced reversal rate, shown are three independent glia-ablated lines (gray symbols). Bars, mean ± SEM, Student’s *t-*test with Bonferroni correction for multiple comparisons to WT (black circles), * *p*<0.05; n=5 movies. Source data are provided as a Source Data file.

**
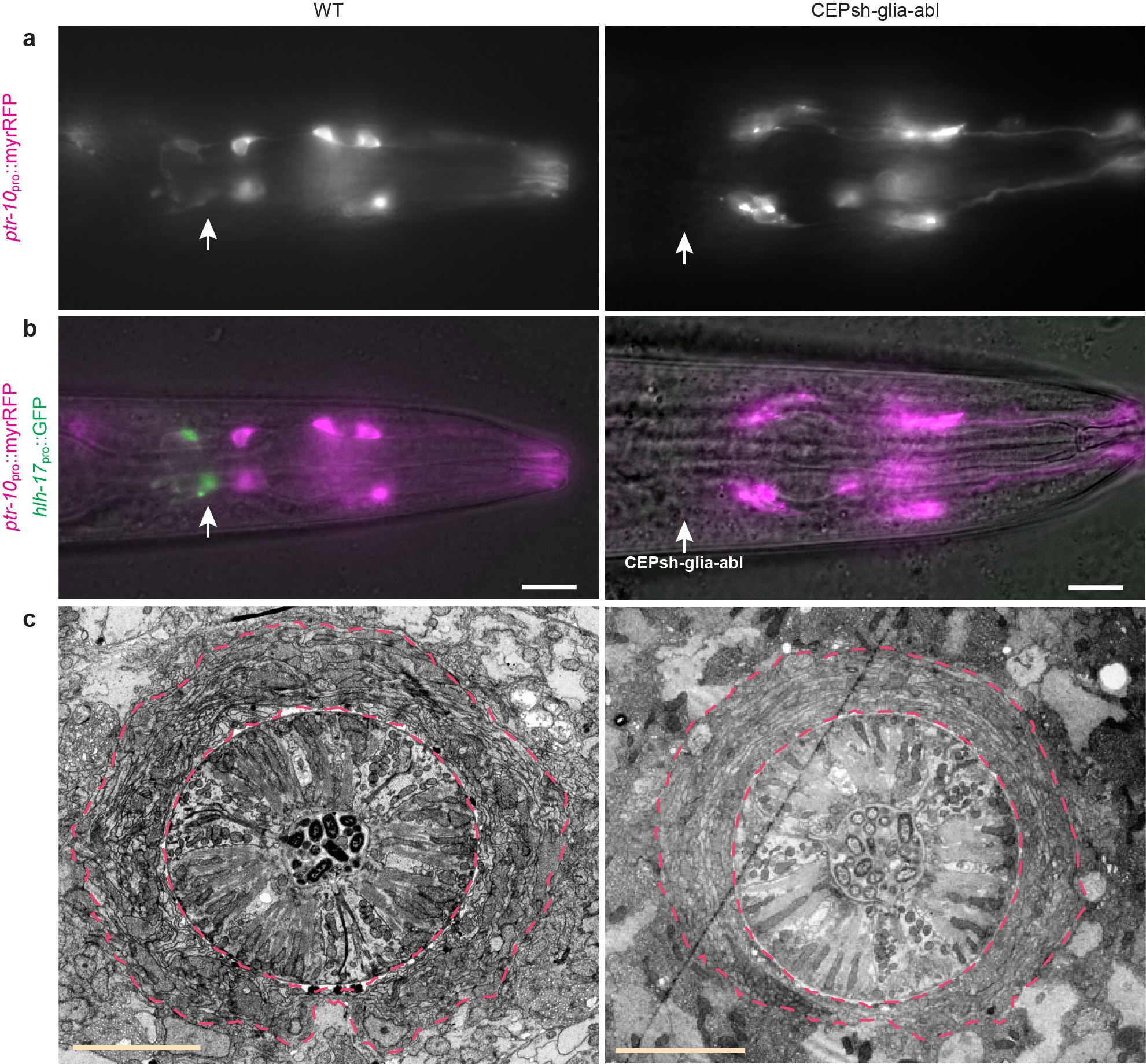
**

**Supplementary Fig. 2. Post-embryonic ablation of CEPsh glia does not affect nerve-ring structure.**

**a,** Fluorescence images of wild-type and CEPsh glia-ablated L4 larvae expressing *ptr-10* _pro_::myrRFP (myristoylated-RFP; many glia) and **b,** merged image with *hlh-17*_pro_::GFP (CEPsh glia-specific) expression. Arrows, CEPsh glia locations. Scale bars, 20 μm. **c,** Electron micrographs of the nerve-ring region of wild-type and CEPsh glia-ablated animals. Red lines, outer and inner nerve-ring borders. Scale bars, 5 μm.

**
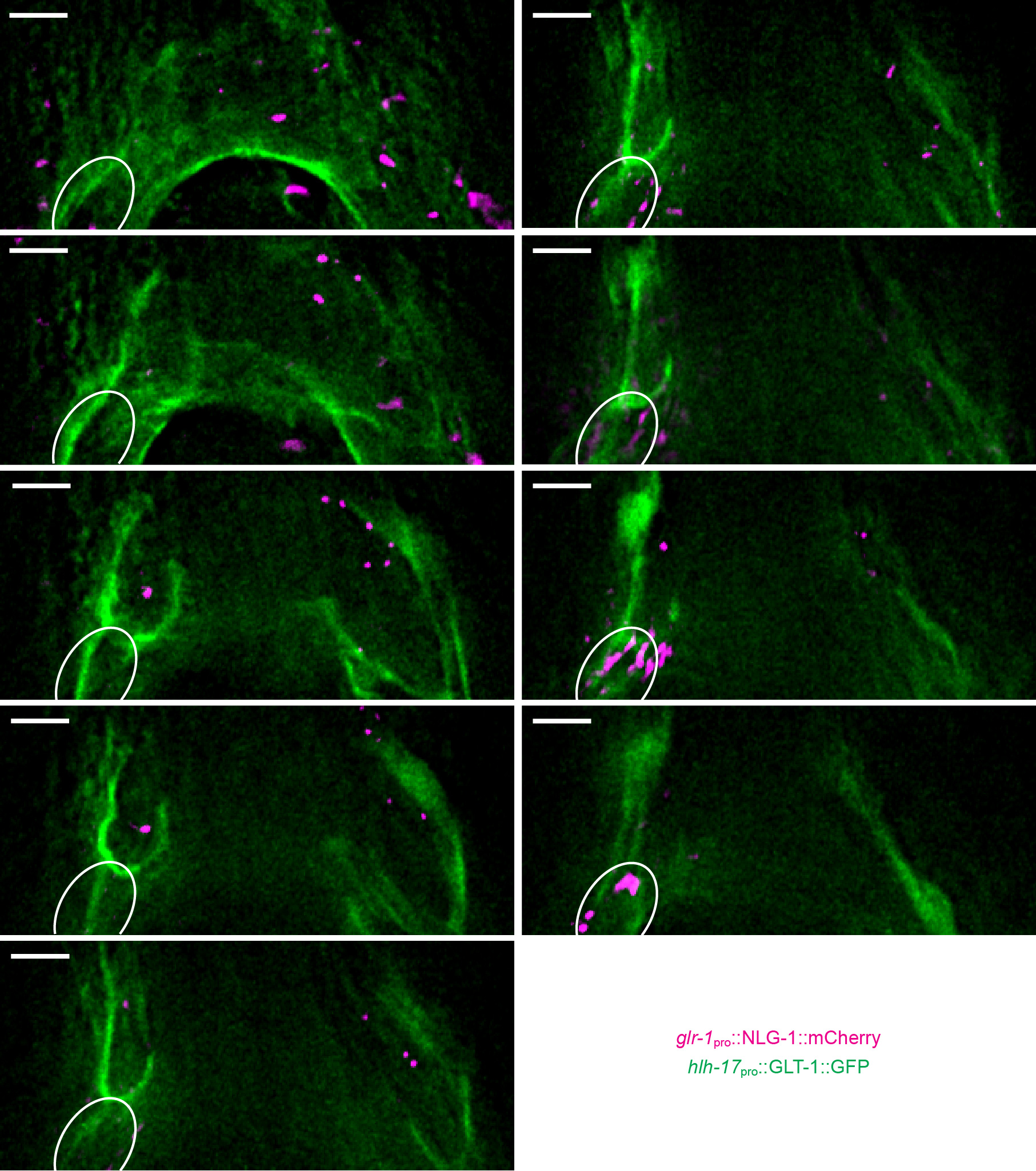
**

**Supplementary Fig. 3. GLT-1 localizes to CEPsh glia plasma membrane and near synapses.**

Adjacent deconvolved optical sections at ~1.4 μm intervals along the *z* imaging *axis*. GLT-1 fused to GFP was expressed in CEPsh glia using the *hlh-17* promoter (green). Synapses are marked by *glr-1*_pro_::NLG-1::mCherry (magenta). Scale bars, 4 μm. White oval, neuronal cell body location.**
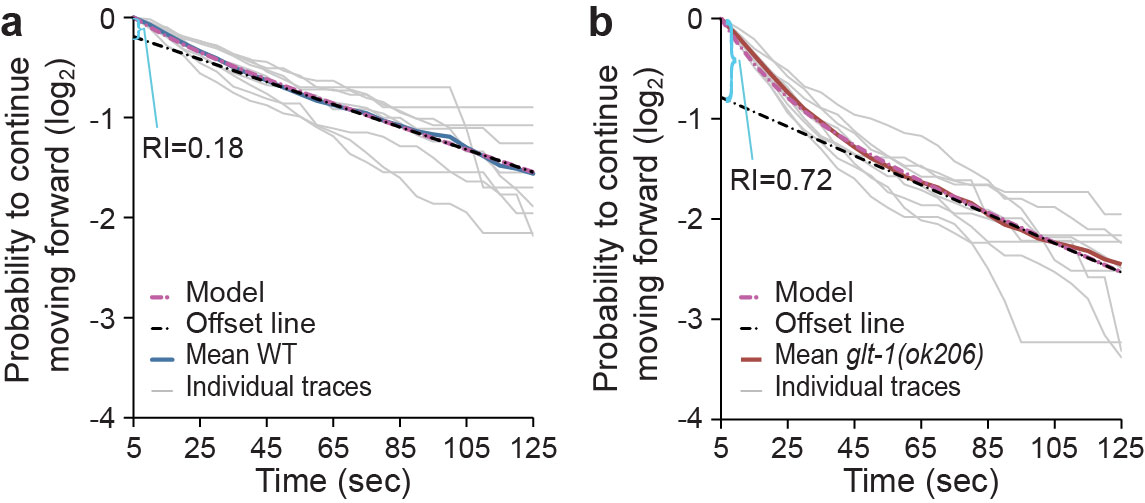
**

**Supplementary Fig. 4. Measurement of repetitive behavior.**

**a,b,** A time-dependent perturbation model (dashed magenta line) can explain repetitive behavior of *glt-1* mutants. At long times, $\log p_{f}\left( t \right)\approx-r_{0}t-\delta l$ (offset, dashed black line), allowing calculation of the repetitive behavior index (RI*=δl/*log2) from the vertical axis intercept (indicated by a cyan bracket) for wild-type (**a**) and *glt-1(ok206)* mutants (**b**)(n=9 movies). Source data are provided as a Source Data file.

**
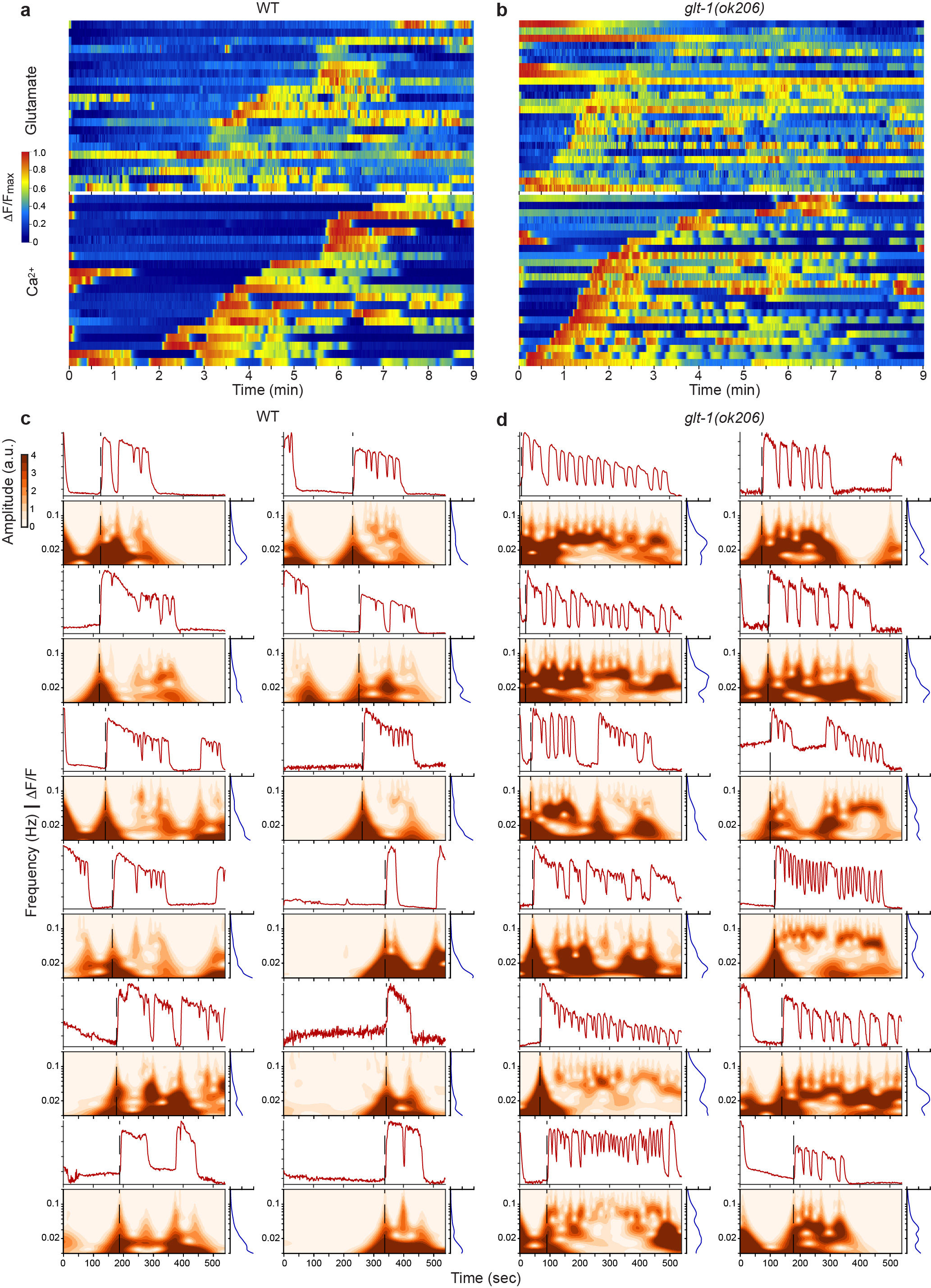
**

**Supplementary Fig. 5. GLT-1 inhibits oscillations in glutamate secretion and AVA activation.**

**a, b,** Heat map plots of relative synaptic glutamate levels at AVA synapses (top) and of relative calcium levels in AVA soma (bottom) in (**a)** wild-type (WT) animals and (**b**) *glt-1(ok206)* mutants. Each row represents recordings of an individual animal. **c, d,** Additional plots, as presented in Fig. 4 a-f, indicating spontaneous calcium dynamics in AVA neuron (top), time-dependent frequency spectrum (bottom left), and time-averaged frequency amplitudes (bottom right) in (**c)** wild-type (WT) animals and (**d**) *glt-1(ok206)* mutants. Source data are provided as a Source Data file.**
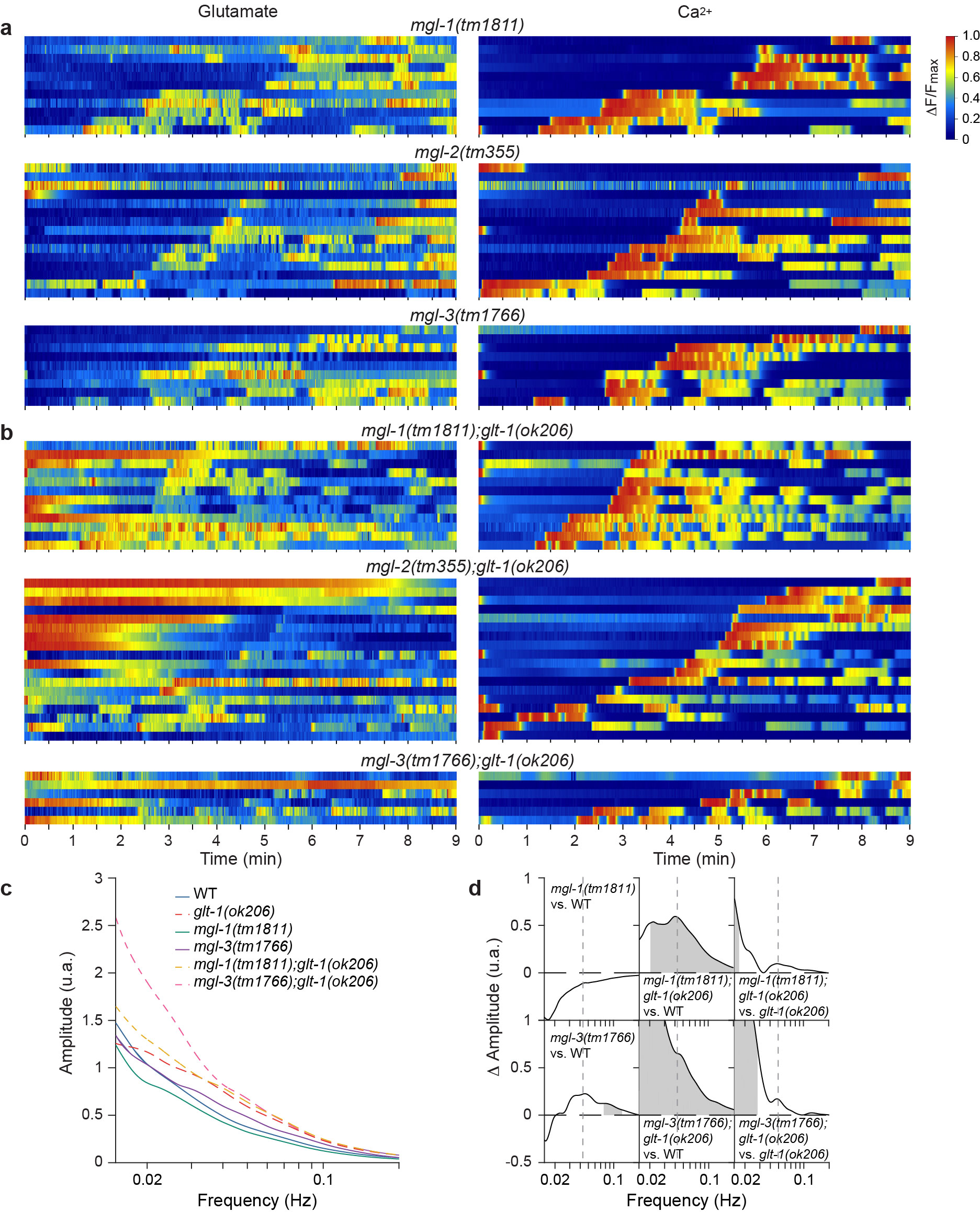
**

**Supplementary Fig. 6. Oscillations induced in *glt-1* mutants do not require MGL-1 or MGL-3.**

**a, b,** Heat map plots for indicated genotypes of relative synaptic glutamate levels at AVA synapses (left) and of relative calcium levels in AVA soma (right) in (**a**) wild-type (WT) and (**b**) *glt-1(ok206)* mutant background. Each line represents recordings of an individual animal. **c,** Mean time-averaged frequency amplitudes for the indicated strains based on the traces presented in **a, b**. **d,** Graphs representing the differences in frequency amplitudes between the indicated strain-pairs. Gray shedding, areas of significant difference *p*<0.01, permutation test from bootstrapped ensembles, n=10^4^ bootstrap samples. Source data are provided as a Source Data file.**
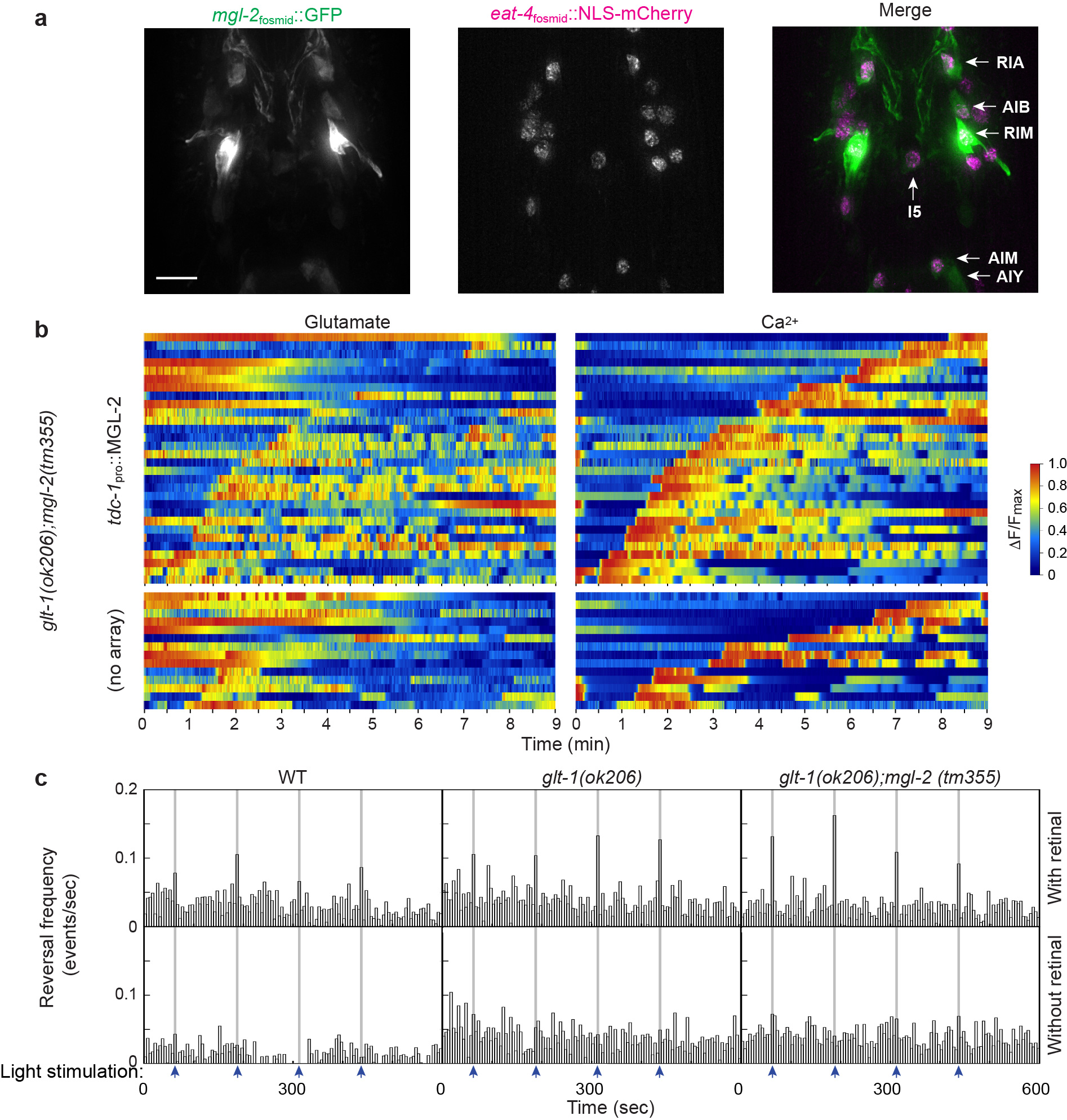
**

**Supplementary Fig. 7. MGL-2 functions in neurons presynaptic to AVA to induce glutamate oscillations and repetitive reversals.**

**a,** Expression of *mgl-2,* maximum intensity projections of deconvolved optical sections (*mgl-2* expressing cells, left; glutamatergic neurons, middle; merge, right). Arrows, identified *mgl-2-*expressing neurons. Scale bars, 8 μm. **b,** Heat map plots of relative synaptic glutamate levels at AVA synapses (left) and of relative calcium levels in AVA soma (right) in *glt-1(ok206); mgl-2(tm355)* mutants with (top) or without (bottom) MGL-2 rescue array in RIM. Each row represents recordings of an individual animal. Note the high background in calcium recordings, due to array expression. **c,** Histograms representing reversal frequencies, binned into 5 seconds intervals, for the indicated strains. Blue arrows and gray shading indicate blue light stimulations; top row, animals treated with retinal; bottom row, without retinal. Source data are provided as a Source Data file.


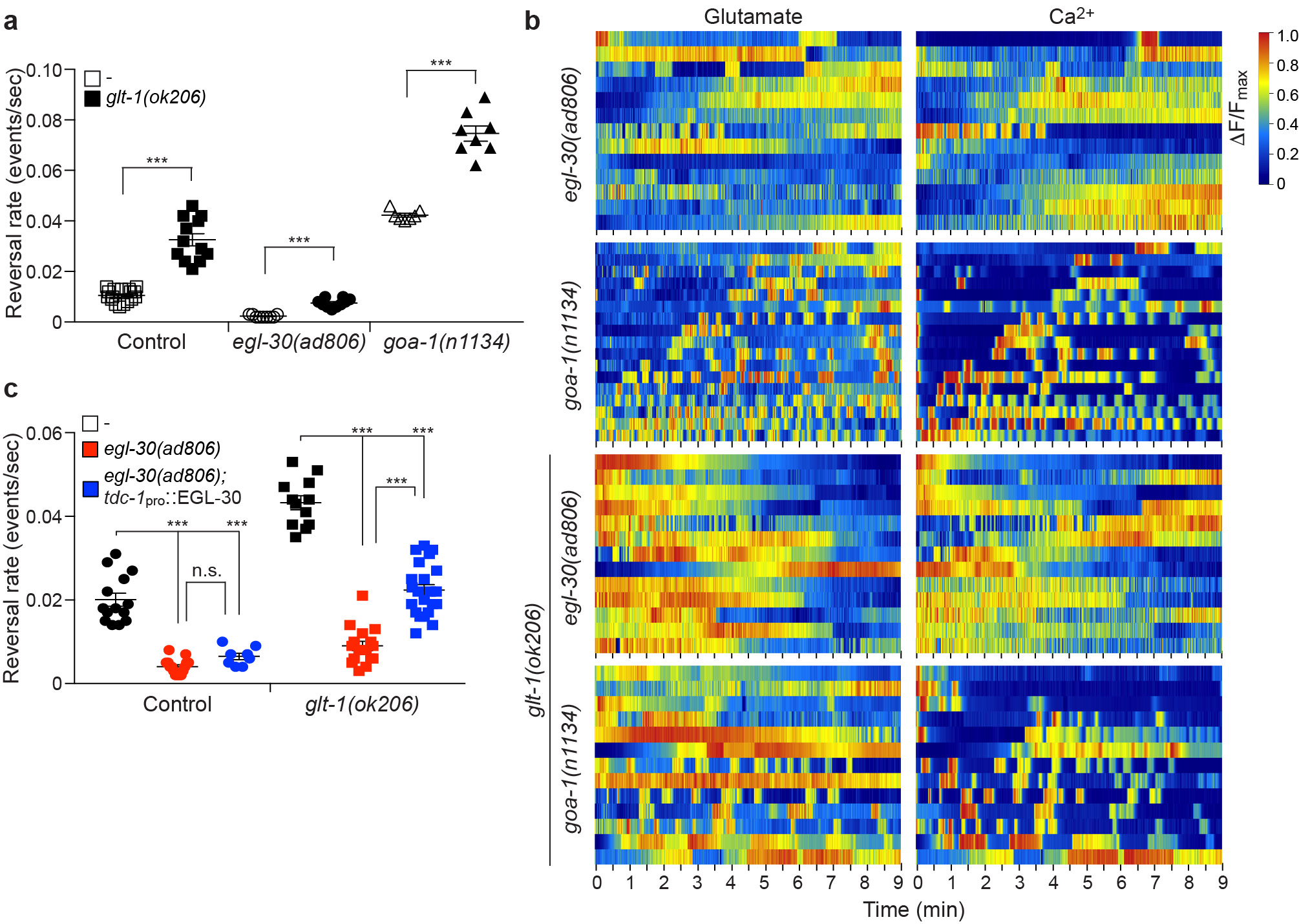


**Supplementary Fig. 8. Glutamate spillover-induced hyper-reversals and glutamate oscillations are mediated by EGL-30/Gαq.**

**a,** Reversal rates in control (open boxes; n=17 movies), *glt-1(ok206)* (black boxes; n=12 movies), *egl-30(ad806)* (open circles; n=9 movies)*, egl-30(ad806); glt-1(ok206)* (black circles; n=10 movies)*, goa-1(n1134)* (open triangles; n=7 movies) and *goa-1(n1134); glt-1(ok206)* (black triangles; n=8 movies) strains (Student’s *t-*test comparing each Gα protein mutant alone to its *glt-1* double mutant, *** *p*<0.0005). **b,** Heat map plots for indicated genotypes of relative synaptic glutamate levels at AVA synapses (left) and of relative calcium levels in AVA soma (right). Each row represents recordings of an individual animal. **c,** Reversal rates in control (black circles; n=14), *egl-30(ad806)* (red circles; n=15 movies)*, egl-30(ad806); tdc-1*_pro_::EGL-30 (blue circles; n=8 movies), *glt-1(ok206)* (black boxes; n=12 movies), *egl-30(ad806); glt-1(ok206)* (red boxes; n=16 movies) and *egl-30(ad806); glt-1(ok206); tdc-1*_pro_::EGL-30 (blue boxes; n=22 movies) strains (ANOVA Tukey’s HSD post hoc test, n.s., non-significant, *** *p*<0.0005; n≥8 movies). **(a, c)** Bars, mean ± SEM. Source data are provided as a Source Data file.

**Supplementary Table**

| Head neurons | Presynaptic to AVA (number of total synapses) | Glutamatergic neuron  (*eat-4* expression) |
| --- | --- | --- |
| RIM | 11 | + |
| ADA | 9 | + |
| AIB | 5 | + |
| RIA | 4 | + |
| AIM | 1 | + |
| RIG | - | + |
| AIY | - | - |
| Pharyngeal neurons |  |  |
| I2 |  | + |
| M3 |  | + |
| I5 |  | +/- |

**Supplementary Table 1. *mgl-2-*expressing neurons.**

Presynaptic contact with AVA taken from the WormWiring webpage (<http://wormwiring.org>).
