# Supplementary material for: Glutamate spillover in C. elegans triggers repetitive behavior through presynaptic activation of MGL-2/mGluR5
Source: Nat Commun. 2019 Apr 23;10:1882. doi: 10.1038/s41467-019-09581-4 (PMC6478929; doi:10.1038/s41467-019-09581-4)
Supplement: Supplementary file 3 — Reporting Summary [file 41467_2019_9581_MOESM3_ESM.pdf]

nature research

Corresponding author(s): Shai Shaham

Reporting Summary

Nature Research wishes to improve the reproducibility of the work that we publish. This form provides structure for consistency and transparency in reporting. For further information on Nature Research policies, see Authors & Referees and the Editorial Policy Checklist.

Statistical parameters

When statistical analyses are reported, confirm that the following items are present in the relevant location (e.g. figure legend, table legend, main text, or Methods section).

n/a	Confirmed	
☐	☒	The exact sample size (*n*) for each experimental group/condition, given as a discrete number and unit of measurement
☐	☒	An indication of whether measurements were taken from distinct samples or whether the same sample was measured repeatedly
☐	☒	The statistical test(s) used AND whether they are one- or two-sided<br>*Only common tests should be described solely by name; describe more complex techniques in the Methods section.*
☒	☐	A description of all covariates tested
☐	☒	A description of any assumptions or corrections, such as tests of normality and adjustment for multiple comparisons
☐	☒	A full description of the statistics including central tendency (e.g. means) or other basic estimates (e.g. regression coefficient) AND variation (e.g. standard deviation) or associated estimates of uncertainty (e.g. confidence intervals)
☐	☒	For null hypothesis testing, the test statistic (e.g. *F*, *t*, *r*) with confidence intervals, effect sizes, degrees of freedom and *P* value noted<br>*Give P values as exact values whenever suitable.*
☒	☐	For Bayesian analysis, information on the choice of priors and Markov chain Monte Carlo settings
☒	☐	For hierarchical and complex designs, identification of the appropriate level for tests and full reporting of outcomes
☒	☐	Estimates of effect sizes (e.g. Cohen's *d*, Pearson's *r*), indicating how they were calculated
☐	☒	Clearly defined error bars<br>*State explicitly what error bars represent (e.g. SD, SE, CI)*

Our web collection on statistics for biologists may be useful.

Software and code

Policy information about availability of computer code

Data collection	Movies of animals locomotion was recorded using the Pylon viewer (Basler) software. Calcium and glutamate recordings were done using the AxioVision (Ziess) software. RNA sequencing was done using the HiSeq2000 sequencer and Fastq files were generated using CASAVA v1.8.2 (illumina) followed by alignment using STAR v2.3.
Data analysis	Analysis of worm locomotion was done using a custom Java script (described in the method section). Analysis of glutamate and calcium dynamics were done using a custom Matlab script (described in the method section). RNA differential expression analysis was done using DESeq2 code. Hierarchical clustering was done using an R code. Over representation analysis was done using a castum R code (described in the method section).

For manuscripts utilizing custom algorithms or software that are central to the research but not yet described in published literature, software must be made available to editors/reviewers upon request. We strongly encourage code deposition in a community repository (e.g. GitHub). See the Nature Research guidelines for submitting code & software for further information.

Data

Policy information about availability of data

All manuscripts must include a data availability statement. This statement should provide the following information, where applicable:

- Accession codes, unique identifiers, or web links for publicly available datasets
- A list of figures that have associated raw data
- A description of any restrictions on data availability

The RNA-seq data sets generated during the current study have been deposited in the European Nucleotide Archive (ENA) with the primary accession code PRJEB31134.
The source data underlying Figs 2a,c, 3a-f, 4i, 5a-c, 6a,b, and Supplementary Figs 1e, 4a,b, 5a,b, 6a,b, 7b, 8a-c are provided as a Source Data file. All customized codes are available from the corresponding author upon request. A reporting summary is provided for this article is available as a Supplementary Information file.

Field-specific reporting

Please select the best fit for your research. If you are not sure, read the appropriate sections before making your selection.

☒ Life sciences ☐ Behavioural & social sciences ☐ Ecological, evolutionary & environmental sciences

For a reference copy of the document with all sections, see nature.com/authors/policies/ReportingSummary-flat.pdf

Life sciences study design

All studies must disclose on these points even when the disclosure is negative.

Sample size	The sample size and statistical tests were chosen based on previous studies with similar methodologies and the data met the assumptions for each statistical test performed. No statistical method was used in deciding sample sizes.
Data exclusions	For behavior assays, before initiating the locomotion analysis, the movies were inspected by eye for damaged or dead animals, those animals were excluded from the analysis. For Ca/glutamate imaging, animals that showed strong movement were excluded since the ROI couldn't be reliably defined.
Replication	All data presented in the manuscript represent the mean of multiple measurements that were acquired over different days.
Randomization	Samples were allocated to groups of the genetic background (genotype), detected by standard genetic/ genomic approaches. Otherwise samples were randomly selected within these groups, based on previous studies with similar methodologies.
Blinding	Locomotion and Ca/glutamate dynamic analyses were done using a computer code. Reversal responses to mechanical stimuli were done manually, and hence all scoring were done blindly, such that the investigator is not aware of which strain is being scored.

Reporting for specific materials, systems and methods

Materials & experimental systems

n/a	Involved in the study
☒	☐ Unique biological materials
☒	☐ Antibodies
☒	☐ Eukaryotic cell lines
☒	☐ Palaeontology
☐	☒ Animals and other organisms
☒	☐ Human research participants

Methods

n/a	Involved in the study
☒	☐ ChIP-seq
☒	☐ Flow cytometry
☒	☐ MRI-based neuroimaging

Animals and other organisms

Policy information about studies involving animals; ARRIVE guidelines recommended for reporting animal research

Laboratory animals	All C. elegans strains that were generated for this study are described in the method section and are available from our laboratory.
Wild animals	This study did not involve wild animals.

Field-collected samples	This study did not involve animals collected from the field.
