# Supplementary material for: Glutamate spillover in C. elegans triggers repetitive behavior through presynaptic activation of MGL-2/mGluR5
Source: Nat Commun. 2019 Apr 23;10:1882. doi: 10.1038/s41467-019-09581-4 (PMC6478929; doi:10.1038/s41467-019-09581-4)
Supplement: Supplementary file 4 — Description of Additional Supplementary Files [file 41467_2019_9581_MOESM4_ESM.pdf]

Supplementary data legends:

Supplementary Data 1. Enrichment of cell-type-specific mouse brain-cell gene homologs in CEPsh glia.
a, No. observed CEPsh glia-expressed genes with orthologs in the various mouse glial cells (gene names are indicated in panel **b**) at different FE (fold enrichment) cutoff values are in green. No. predicted CEPsh glia-expressed genes with orthologs in mouse glial cells at different FE cutoff values (see online Methods) are in gray. Gene expression association of the mouse glia with CEPsh glia at the different FE cutoffs are indicated in the columns shaded in blue. **b,** Mouse gene names indicted in column A. Enrichment in mouse brain cells indicated in columns B-F. CEPsh glia genes indicated in column G and enrichment indicated in column H. Columns I,J, mouse genes MGI and Uniprot accession numbers. Column K, *C. elegans* WormBase accession numbers.

Supplementary Movie legends:
Supplementary Movie 1. Locomotory behavior of wild-type animals.
Wild-type animals moving on an agar plate in the absence of food. Images taken at 2 fps, and presented at 5X speed. Forward locomotion, green. Backward, red. Blue indicates a turn. A magenta circle highlights reversal onset.

Supplementary Movie 2. Locomotory behavior of CEPsh glia-ablated animals.
As in Movie 1, CEPsh glia-ablated animals.

Supplementary Movie 3. Locomotory behavior of *glt-1(ok206)* mutant animals.
As in Movie 1, *glt-1(ok206)* animals.

Supplementary Movie 4. GLT-1 localizes to CEPsh glia plasma membrane and near synapses.
3D reconstruction of deconvolved contiguous optical sections. Green, GLT-1(cDNA) fused to GFP expressed in CEPsh glia using the *hlh-17* promoter. Red, neuroligin (cDNA) fused to mCherry expressed in neurons using the *glr-1* promoter marking synapses within the nerve ring.

Supplementary Movie 5. Reversal response to a mechanical stimulus in a wild-type animal.
Images taken at 5 fps, and presented at 4X speed.

Supplementary Movie 6. Reversal response to a mechanical stimulus in a *glt-1(ok206)* mutant.
As in Movie 5, for a *glt-1(ok206)* mutant animal. Note the multiple reversal events of various durations following a single stimulus.

Supplementary Movie 7. Simultaneous recordings of extracellular glutamate and AVA calcium activity in a wild-type animal.
Extracellular glutamate and AVA calcium dynamics recorded for 9 minutes from animal expressing iGluSnFR and RCaMP in AVA. Images captured at 1 fps, movie played at 30X speed. Glutamate signal (left) recorded form AVA process (ROI indicated by white line), and calcium (right) recorded from AVA soma (indicated by an arrow).

Supplementary Movie 8. Simultaneous recordings of extracellular glutamate and AVA calcium activity in a *glt-1(ok206)* mutant.
As in Movie 7, *glt-1(ok206)* mutant.
